# Neighborhood Income Mobility and Risk of Neonatal and Maternal Morbidity

**DOI:** 10.1001/jamanetworkopen.2023.15301

**Published:** 2023-05-23

**Authors:** Jennifer A. Jairam, Simone N. Vigod, Arjumand Siddiqi, Jun Guan, Alexa Boblitz, Xuesong Wang, Patricia O’Campo, Joel G. Ray

**Affiliations:** 1 Division of Epidemiology, Dalla Lana School of Public Health, University of Toronto, Toronto, Ontario, Canada; 2 MAP Centre for Urban Health Solutions, St Michael's Hospital, Toronto, Ontario, Canada; 3 Department of Psychiatry, University of Toronto, Toronto, Ontario, Canada; 4 Institute of Health Policy, Management and Evaluation, University of Toronto, Toronto, Ontario, Canada; 5 ICES, Toronto, Ontario, Canada; 6 Women's College Hospital, Toronto, Ontario, Canada; 7 Gillings School of Global Public Health, University of North Carolina at Chapel Hill; 8 Department of Social and Behavioral Sciences, Harvard T.H. Chan School of Public Health, Boston, Massachusetts; 9 Keenan Research Centre, St Michael’s Hospital, Toronto, Ontario, Canada; 10 Department of Obstetrics and Gynecology, St Michael’s Hospital, Toronto, Ontario, Canada

## Abstract

**Importance:**

Residing in a low-income neighborhood is generally associated with worse pregnancy outcomes. It is not known if moving from a low- to higher-income area between 2 pregnancies alters the risk of adverse birth outcomes in the subsequent birth compared with women who remain in low-income areas for both births.

**Objective:**

To compare the risk of adverse maternal and newborn outcomes among women who achieved upward area-level income mobility vs those who did not.

**Design, Setting, and Participants:**

This population-based cohort study was completed in Ontario, Canada, from 2002 to 2019, where there is universal health care. Included were all nulliparous women with a first-time singleton birth at 20 to 42 weeks’ gestation, each residing in a low-income urban neighborhood at the time of the first birth. All women were then assessed at their second birth. Statistical analysis was conducted from August 2022 to April 2023.

**Exposure:**

Movement from a lowest-income quintile (Q1) neighborhood to any higher-income quintile neighborhood (Q2-Q5) between the first and second birth.

**Main Outcomes and Measures:**

The maternal outcome was severe maternal morbidity or mortality (SMM-M) at the second birth hospitalization or up to 42 days post partum. The primary perinatal outcome was severe neonatal morbidity or mortality (SNM-M) within 27 days of the second birth. Relative risks (aRR) and absolute risk differences (aARD) were estimated by adjusting for maternal and infant characteristics.

**Results:**

A total of 42 208 (44.1%) women (mean [SD] age at second birth, 30.0 [5.2] years) experienced upward area-level income mobility, and 53 409 (55.9%) women (age at second birth, 29.0 [5.4] years) remained in income Q1 between births. Relative to women who remained in income Q1 between births, those with upward mobility had a lower associated risk of SMM-M (12.0 vs 13.3 per 1000 births), with an aRR of 0.86 (95% CI, 0.78 to 0.93) and aARD of −2.09 per 1000 (95% CI, −3.1 to −0.9 per 1000 ). Likewise, their newborns experienced lower respective rates of SNM-M (48.0 vs 50.9 per 1000 live births), with an aRR of 0.91 (95% CI, 0.87 to 0.95) and aARD of −4.7 per 1000 (95% CI, −6.8 to −2.6 per 1000).

**Conclusions and Relevance:**

In this cohort study of nulliparous women living in low-income areas, those who moved to a higher-income area between births experienced less morbidity and death in their second pregnancy, as did their newborns, compared with those who remained in low-income areas between births. Research is needed to determine whether financial incentives or enhancement of neighborhood factors can reduce adverse maternal and perinatal outcomes.

## Key Points

**Question:**

Do adverse birth outcomes differ among women who move from a low- to higher-income neighborhood between births?

**Findings:**

This population-based cohort study included 95 617 women residing in a low-income neighborhood at their first birth, and all had universal health care. Women who moved to a higher-income neighborhood between births had lower rates of severe maternal morbidity/mortality than those who remained in a low-income neighborhood between births (12.0 vs 13.3 per 1000 births), and their newborns experienced lower rates of severe neonatal morbidity/mortality (48.0 vs 50.9 per 1000 live births).

**Meaning:**

These findings suggest that women who move from a low-income to a higher-income neighborhood between births experience less morbidity and death, as do their newborns.

## Teaser

This cohort study evaluates the association of neighborhood income mobility and risk of neonatal and maternal morbidity among patients in Ontario, Canada.

## Introduction

Socioeconomic position is a determinant of maternal and infant birth outcomes.^1^ Women of low socioeconomic position tend to have worse health outcomes during pregnancy than those with greater socioeconomic stability.^1,2,3,4,5,6,7,8,9^ A growing body of epidemiological studies suggests that maternal and newborn outcomes are shaped by area-level measures of socioeconomic position, independent of individual-level attributes.^2,3,4,5,7,8,10,11,12^ Residing in an economically deprived area limits the availability, access, and quality of goods, education, and health and social services.^12,13,14,15^

Neighborhood income characterizes the economic profile of a local geographic area. Prior research on neighborhood income and adverse pregnancy outcomes generally focused on indirect proxies of newborn risk, such as preterm birth, small or large-for-gestational-age birth weight, and a low Apgar score,^3,5^ rather than hard outcomes such as severe neonatal morbidity and/or neonatal death. Moreover, they tended to compare women of low- and high-income groups,^16^ rather than being confined to women living in low-income areas, namely, those at highest risk for maternal and neonatal morbidity and death. Existing research also largely focused on income mobility between a woman’s early life and that measured in adulthood,^17,18,19^ rather than a change in her income position exclusively within her reproductive years, which may provide insight on the mutability of risk between pregnancies, while offering a key period for prevention and intervention. Identifying women and newborns at higher risk of morbidity cannot only impact their health trajectories, including their risk of disability and death, but has well-defined economic, emotional, and social consequences for their families and the local health care system.^20^

The current study builds on previous research in which we found that women residing in the lowest-income neighborhoods are at highest risk of having a newborn with concomitant preterm and severe small-for-gestational-age birth weight.^21^ Findings from our other studies suggest that immigrant women and their newborns have an overall lower risk of severe maternal morbidity or maternal mortality and severe neonatal morbidity and neonatal mortality, respectively, compared with Canadian-born women and their newborns, all of whom were residing in low-income neighborhoods.^22,23^ In the current study, comprising mothers living in low-income urban neighborhoods, we evaluated their subsequent risk of adverse maternal and newborn outcomes, while comparing those who achieved upward area-level income mobility between 2 consecutive births vs those who did not experience any upward income mobility.

## Methods

### Study Design, Setting, and Participants

This retrospective population-based cohort study was conducted in Ontario, Canada’s most populous and ethnically diverse province, with universal health care insurance available to all permanent residents. Included were all nulliparous women who had 2 consecutive singleton hospital-based live births or stillbirths at 20 to 42 weeks’ gestation. The study was restricted to those residing in an urban low-income quintile (Q1) neighborhood at the time of the first birth, aged 15 to 50 years, and who had a valid Ontario Health Insurance Plan number at each birth hospitalizations (eTable 1 and eFigure in Supplement 1).

This study followed the Strengthening the Reporting of Observational Studies in Epidemiology (STROBE) reporting guideline. Ethics approval was received from the Health Sciences Research Ethics Board at the University of Toronto.

### Data Sources

We used administrative data at ICES, an independent, nonprofit research institute. Ontario’s health information privacy law allows ICES to collect and analyze health care and demographic data without consent for health system evaluation and improvement. These data sets are a valid and reliable source for conducting perinatal research,^24,25,26,27,28^ and include information about vital status, sociodemographic factors, hospitalizations, census data, physician claims, and immigration (eTable 2 in Supplement 1). All hospital live births and stillbirths were identified in the ICES MOMBABY database, which captures about 98% of all births in Ontario, and links inpatient admission records of delivering mothers and their newborns (eTable 2 in Supplement 1). Data sets were linked using unique encoded identifiers and analyzed at ICES. Maternal immigrant status was determined using the Immigration, Refugee and Citizenship Canada Permanent Residents database (eTable 2 in Supplement 1).

### Study Exposures

The primary exposure was any upward mobility, from an urban income Q1 area at the first birth to any higher-income (Q2 to Q5) urban area at the second birth, which was compared with remaining in an urban income Q1 neighborhood at both births (eTable 1 in Supplement 1). Neighborhood income quartile is based on the average income per single-person equivalent in a dissemination area, adjusted for household size,^29^ and obtained from census area-level income data.^30^ A dissemination area is the smallest unit of census geography containing between 400 to 700 people,^31^ and follows natural urban boundaries and census divisions.^32^ The Statistics Canada Postal Code Conversion File Plus was used to link a woman’s residential postal code at the time of her obstetrical delivery to a dissemination area in the Canadian census year closest to her hospitalization date.^30^ The neighborhood income quartile was then assigned to a woman at her first birth, and updated at her second birth.

A secondary exposure was degree of upward mobility between the first and second birth, categorized into 3 mobility patterns: (1) a Q1 to Q2 or Q3 neighborhood, (2) a Q1 to Q4 or Q5 neighborhood, each relative to (3) remaining in a Q1 neighborhood at both births.

### Outcomes

The primary maternal outcome was a composite of severe maternal morbidity (SMM) or all-cause maternal mortality (SMM-M) in the second pregnancy. SMM is a near-miss proxy that is associated with maternal death and prolonged hospital length of stay.^33,34,35^ The SMM measure was developed and validated by the Canadian Perinatal Health Surveillance System,^34,36^ and includes 40 unique indicators using the *International Statistical Classification of Diseases and Related Health Problems, Tenth Revision, Canada (ICD-10-CA)* diagnostic codes^37^ and the *Canadian Classification of Health Interventions (CCI)*^38^ procedural codes. Examples of SMM indicators are maternal ICU admission, severe preeclampsia, and use of assisted ventilation (eTable 1 in Supplement 1). SMM-M was identified during a woman’s second birth hospitalization, or up to 42 days thereafter.

The primary newborn outcome was a composite of severe neonatal morbidity (SNM) or all-cause neonatal mortality (SNM-M) in the second pregnancy. SNM is a near-miss proxy that is associated with neonatal death and hospital readmission among infants in their first year of life.^39^ SNM-M was based on the English-version Neonatal Adverse Outcomes Indicator, a validated measure consisting of 23 components, 16 neonatal diagnoses and 7 procedures that relate to different body systems,^39^ and recorded using *ICD-10-CA* diagnostic codes and *CCI* procedural codes. Examples of SNM-M indicators include neonatal death, newborn seizure, newborn resuscitation, and ventilatory support (eTable 1 in Supplement 1).^39^ SNM-M was identified during the birth admission for the second infant or up to 27 days thereafter.^39^ A secondary perinatal outcome was preterm birth (PTB) live birth or stillbirth arising before 37 weeks’ gestation, again, occurring in the second pregnancy.

### Statistical Analysis

Means and proportions were assessed using standardized differences, contrasting women with any upward mobility vs no upward mobility. A standardized difference value of >0.10 was considered to be clinically important.^40^ The top 20 most common SMM and SNM indicators in the second pregnancy were also presented, irrespective of neighborhood income mobility between pregnancies.

For each binary study outcome, women with any upward mobility were compared with those with no upward mobility, using an adapted approach to logistic regression analysis by Austin^41^ to estimate relative risks (RR) and absolute risk differences (ARD). This approach is based on marginal probabilities of the outcome of interest, called population-average (mean) probabilities of outcomes for exposed and unexposed study participants. The 95% CIs of RR and ARD were estimated by bootstrap method with resampling 1500 replicates.^41^ The same modeling approach was also used to assess each outcome by the degree of upward mobility, namely, from Q1 to Q2 or Q3, or Q1 to Q4 or Q5, each relative to no upward mobility between pregnancies. All RRs and ARDs were adjusted for maternal age at the second birth hospitalization (15-19, 20-29, 30-39, and 40-50 years); birth interval (<18, 18-36, 37-60, 61-119, and ≥120 months); maternal world region of origin (Canada, Caribbean, East Asia and Pacific, Latin America, Middle East and North Africa, South Asia, Sub-Saharan Africa, and Western Nations and Europe)^42,43^; prepregnancy hypertension within 1 to 365 days before the second birth hospitalization; prepregnancy diabetes within 1 to 365 days before the second birth hospitalization and gestational diabetes identified at the second birth hospitalization. For the outcome of SNM-M, RRs and ARDs were further adjusted for any structural congenital anomaly diagnosed in the second infant’s birth hospitalization.

Immigrants may have a different health trajectory and pregnancy outcomes than nonimmigrants.^22,23,44,45^ Accordingly, an additional analysis was completed for the corresponding main model of any upward income mobility and SMM-M or SNM-M. Therein, the interaction term of “neighborhood income mobility between births and immigrant status” was added to each respective model. Even so, it was decided, a priori, that adjusted RRs (aRRs) and adjusted ARDs (aARD) would be shown for these 2 groups, irrespective of the statistical significance of the interaction terms.

Statistical analysis was conducted from August 2022 to April 2023. All analyses were performed using SAS version 9.4 (SAS Institute Inc). Statistical significance was set at *P* < .05, and all tests were 2-tailed.

## Results

There were 1 403 386 births during the study period. Of these, the final cohort comprised 95 617 nulliparous women residing in an urban low-income Q1 neighborhood at their first birth, and who had a subsequent birth during the study period (eFigure in Supplement 1). Of these, 99.9% had a live birth and 0.01% a stillbirth (Table 1). There were 42 208 women (44.1%) who experienced any upward mobility between births, and 53 409 (55.9%) women who had no upward mobility between births (Table 1).

**Table 1.** Maternal and Birth Characteristics of Study Participants by Neighborhood Income Quintile (Q) Upward Mobility Pattern Between 2 Consecutive Births^a^

Characteristic	Participants, No. (%)		Standardized difference^d^
	Any upward mobility: moved from a Q1 neighborhood to any higher Q2-Q5 neighborhood between births (n = 42 208)^b^	No upward mobility: resided in a Q1 neighborhood at both births (n = 53 409)^c^	
Maternal			
Age at second birth, mean (SD), y	30.0 (5.2)	29.0 (5.4)	0.18
15-19	668 (1.6)	1467 (2.7)	0.08
20-29	18 493 (43.8)	27 133 (50.8)	0.14
30-39	21 830 (51.7)	23 498 (44.0)	0.16
40-50	1217 (2.9)	1311 (2.5)	0.03
Time interval between first and second births, median (IQR), months	37 (26-56)	30 (21-45)	0.38
<18	2879 (6.8)	7349 (13.8)	0.23
18-36	17 590 (41.7)	26 702 (50.0)	0.17
37-60	12 770 (30.3)	12 539 (23.5)	0.15
61-119	7983 (18.9)	6168 (11.5)	0.21
≥120	986 (2.3)	651 (1.2)	0.09
Prepregnancy hypertension before the second birth	1078 (2.6)	1347 (2.5)	0.00
Prepregnancy diabetes before the second birth or gestational diabetes at the second birth	4030 (9.5)	5452 (10.2)	0.02
Immigrant status			
Nonimmigrant	28 299 (67.0)	33 283 (62.3)	0.10
Immigrant^e^	13 909 (33.0)	20 126 (37.7)	0.10
World region of origin among the immigrant women			
Caribbean	797 (5.7)	1379 (6.9)	0.05
East Asia and Pacific	3827 (27.5)	4499 (22.4)	0.02
Latin America	907 (6.5)	1265 (6.3)	0.02
Middle East and North Africa	1204 (8.7)	1885 (9.4)	0.04
South Asia	4837 (34.8)	7764 (38.6)	0.09
Sub-Saharan Africa	541 (3.9)	1659 (8.2)	0.13
Western Nations and Europe	1796 (12.9)	1675 (8.3)	0.06
Duration of residence in Ontario among the immigrant women at the second birth, median (IQR), y^e^	8 (5-12)	6 (4-11)	0.29
<10	9183 (66.0)	14 591 (72.5)	0.13
≥10	4726 (34.0)	5535 (27.5)	0.03
Newborn from the second pregnancy			
Live birth	42 202 (99.99)	53 403 (99.99)	0.00
Stillbirth	6 (0.01)	6 (0.01)	0.00
Birth weight, mean (SD), g^f^	3389 (544.6)	3356 (551.0)	0.06
250-1499	263 (0.6)	401 (0.8)	0.02
1500-2499	1675 (4.0)	2205 (4.1)	0.01
2500-3999	35 507 (84.1)	45 253 (84.7)	0.02
≥4000	4763 (11.3)	5550 (10.4)	0.03
Gestational age at birth, median (IQR), weeks	39.0 (38-40)	39.0 (38-40)	0.02
<28	123 (0.3)	179 (0.3)	0.01
28-31 weeks, 6 days	186 (0.4)	267 (0.5)	0.01
32-36 weeks, 6 days	2106 (5.0)	2810 (5.3)	0.01
≥37	39 793 (94.3)	50 153 (93.9)	0.02
Any structural congenital anomaly diagnosed in the second birth hospitalization	1871 (4.4)	2401 (4.5)	0.00

^a^ Data are limited to nulliparous women who were initially residing in a lowest income Q1 urban neighborhood at their first birth, and who also had a second birth. Births include all singleton hospital live births or stillbirths at 20 weeks and 0 days gestation to 42 weeks and 0 days gestation in Ontario, Canada, 2002 to 2019.

^b^ Moved from a Q1 neighorhood to any higher Q2-Q5 neighborhood between births.

^c^ Resided in a Q1 neighborhood at both births.

^d^ A standardized difference of >0.10 is considered to be clinically meaningful.

^e^ Nonrefugee immigrants.

^f^ Birth weight categories approximate the definitions of very low birth weight (≤1499 g), low birth weight (≤2499 g), normal birth weight (2500-3999 g), and high birth weight (≥4000 g).

Women with any upward mobility tended to be older than those with no upward mobility (mean [SD] age at second birth, 30.0 [5.2] vs 29.0 [5.4] years), had a longer time interval between births (median [IQR], 37 [26-56] vs 30 [21-45] months), and were more likely to be nonimmigrants (28 299 participants [67%] vs 33 283 participants 62.3%) (Table 1). Additional characteristics are presented in Table 1.

### Severe Maternal Morbidity or Maternal Mortality

Among the top 20 most prevalent SMM indicators in the second pregnancy, postpartum hemorrhage with red cell transfusion or procedures to the uterus or hysterectomy was most the common (3.2 per 1000 births) (eTable 3 in Supplement 1). Women with any upward mobility between births had a lower rate of SMM-M (12.0 per 1000 births) than those with no upward mobility (13.3 per 1000 births), an aRR of 0.86 (95% CI, 0.78 to 0.93) and aARD of −2.0 cases per 1000 births (95% CI, −3.1 to −0.9 cases per 1000 births) (Table 2). The inverse association for SMM-M was largely the same whether a woman moved to a Q2 or Q3, or Q4 or Q5 income area, relative to no upward mobility (Table 3).

**Table 2.** Risk of SMM-M Arising in the Mother’s Birth Hospitalization of the Second Pregnancy, or up to 42 Days Thereafter, Comparing Women Who Moved From a Neighborhood Income Q1 to Any Higher Neighborhood Income (Q2-Q5) Between Their First and Second Consecutive Births (Any Upward Mobility) vs Those With No Upward Mobility^a^

Neighborhood income mobility between births	No. with SMM-M (rate per 1000 births)	Relative risk (95% CI)		Adjusted absolute risk difference per 1000 births (95% CI)^b^^,^^c^
		Unadjusted^b^	Adjusted^b^^,^^c^	
No upward mobility (n = 53 409)	711 (13.3)	1 [Reference]	1 [Reference]	0 [Reference]
Any upward mobility (n = 42 208)	507 (12.0)	0.90 (0.83 to 0.98)	0.86 (0.78 to 0.93)	−2.0 (−3.1 to −0.9)

Abbreviations: Q, quintile; SMM-M, severe maternal morbidity or maternal mortality.

^a^ Data are limited to nulliparous women who were initially residing in a lowest income Q1 neighborhood at their first birth, and who had a second birth also in Ontario. Births include all singleton hospital live births or stillbirths at 20 weeks and 0 days gestation to 42 weeks and 0 days gestation in Ontario, Canada, 2002 to 2019.

^b^ Using an adapted approach to logistic regression.^41^

^c^ Adjustments are described in the Statistical Analysis section.

**Table 3.** Risk of SMM-M Arising in the Mother’s Birth Hospitalization of the Second Pregnancy, or up to 42 Days Thereafter, by Degree of Upward Mobility From a Neighborhood Income Q1 to a Higher Neighborhood Income Quintile (Q2 or Q3; Q4 or Q5) Between the First and Second Consecutive Birth vs Those With No Upward Mobility^a^

Degree of neighborhood income mobility between births	No. with SMM-M (rate per 1000 births)	Relative risk (95% CI)		Adjusted absolute risk difference per 1000 births (95% CI)^b^^,^^c^
		Unadjusted^b^	Adjusted^b^^,^^c^	
No upward mobility (n = 53 409)	711 (13.3)	1 [Reference]	1 [Reference]	0 [Reference]
Moved from a Q1 to a Q2/3 neighborhood (n = 27 735)	331 (11.9)	0.90 (0.81 to 0.99)	0.85 (0.77 to 0.95)	−2.0 (−3.2 to −0.7)
Moved from a Q1 to a Q4/5 neighborhood (n = 14 473)	176 (12.2)	0.91 (0.80 to 1.04)	0.85 (0.74 to 0.96)	−2.1 (−3.6 to −0.5)

Abbreviations: Q, quintile; SMM-M, severe maternal morbidity or maternal mortality.

^a^ Data are limited to nulliparous women who were initially residing in a lowest income Q1 neighborhood at their first birth, and who had a second birth also in Ontario. Births include all singleton hospital live births or stillbirths at 20 weeks and 0 days gestation to 42 weeks and 0 days gestation in Ontario, Canada, 2002 to 2019.

^b^ Using an adapted approach to logistic regression analysis.^41^

^c^ Adjusted for maternal age at the second birth hospitalization (15-19, 20-29, 30-39, and 40-50 years); birth interval (<18, 18-36, 37-60, 61-119, and ≥120 months); maternal world region of origin (Canada, Caribbean, East Asia and Pacific, Latin America, Middle East and North Africa, South Asia, Sub-Saharan Africa, and Western Nations and Europe); prepregnancy hypertension within 1 to 365 days before the second birth hospitalization; prepregnancy diabetes within 1 to 365 days before the second birth hospitalization; and gestational diabetes identified at the second birth hospitalization.

### Severe Neonatal Morbidity or Neonatal Mortality

Among the top 20 most prevalent SNM indicators in the second pregnancy, newborn ventilatory support was the most common (31.9 per 1000 live births) (eTable 3 in Supplement 1). The rate of SNM-M was lower among newborns whose mother had any upward mobility between births (48.0 per 1000 live births) than those whose mother did not (50.9 per 1000 live births), equivalent to an aRR of 0.91 (95% CI, 0.87 to 0.95) and an aARD of −4.7 cases per 1000 live births (95% CI, −6.8 to −2.6 per 1000 live births) (Table 4). This inverse association was especially more pronounced for newborns whose mother moved to a Q4 or Q5 neighborhood (aRR 0.87; 95% CI, 0.81 to 0.93) (Table 5).

**Table 4.** Risk of SNM-M Arising in the Newborn’s Birth Admission of the Second Pregnancy, or up to 27 Days Thereafter, Comparing Newborns Whose Mother Moved From a Neighborhood Income Q1 to Any Higher Neighborhood Income (Q2-Q5) Between Her First and Second Consecutive Births (Any Upward Mobility) vs Those With No Upward Mobility^a^

Neighborhood income mobility between births	No. with SNM-M (rate per 1000 live births)	Relative risk (95% CI)		Adjusted absolute risk difference per 1000 live births (95% CI)^b^^,^^c^
		Unadjusted^b^	Adjusted^b^^,^^c^	
No upward mobility (n = 53 403)	2717 (50.9)	1 [Reference]	1 [Reference]	0 [Reference]
Any upward mobility (n = 42 202)	2025 (48.0)	0.94 (0.91 to 0.98)	0.91 (0.87 to 0.95)	−4.7 (−6.8 to −2.6)

Abbreviations: Q, quintile; SNM-M, severe neonatal morbidity or neonatal mortality.

^a^ Data are limited to nulliparous women who were initially residing in a lowest income Q1 neighborhood at their first birth, and who had a second birth also in Ontario. Births include all singleton hospital live births at 20 weeks and 0 days gestation to 42 weeks and 0 days gestation in Ontario, Canada, 2002 to 2019.

^b^ Using an adapted approach to logistic regression analysis.^41^

^c^ Adjustments described in Statistical Analysis section.

**Table 5.** Risk of SNM-M Arising in the Newborn’s Birth Admission of the Second Pregnancy, or up to 27 Days Thereafter, by Degree of Upward Mobility From a Neighborhood Income Q1 to a Higher Neighborhood Income Quintile (Q2 or Q3; or Q4 or Q5) Between the First and Second Consecutive Birth vs Those With No Upward Mobility^a^

Degree of neighborhood income mobility between births	No. with SNM-M (rate per 1000 live births)	Relative risk (95% CI)		Adjusted absolute risk difference, (per 1000 live births, 95% CI)^b^^,^^c^
		Unadjusted^b^	Adjusted^b^^,^^c^	
No upward mobility (n = 53 403)	2717 (50.9)	1 [Reference]	1 [Reference]	0 [Reference]
Moved from a Q1 to a Q2/3 neighborhood (n = 27 731)	1354 (48.8)	0.96 (0.91 to 1.01)	0.93 (0.88 to 0.97)	−3.8 (−6.0 to −1.0)
Moved from a Q1 to a Q4/5 neighborhood (n = 14 471)	671 (46.4)	0.91 (0.86 to 0.97)	0.87 (0.81 to 0.93)	−6.7 (−9.8 to −3.7)

Abbreviations: Q, quintile; SNM-M, severe neonatal morbidity or neonatal mortality.

^a^ Data are limited to nulliparous women who were initially residing in a lowest income Q1 neighborhood at their first birth, and who had a second birth also in Ontario. Births include all singleton hospital live births at 20 weeks and 0 days gestation to 42 weeks and 0 days gestation in Ontario, Canada, 2002 to 2019.

^b^ Using an adapted approach to logistic regression analysis.^41^

^c^ Adjustment described in the Statistical Analysis section.

### Preterm Birth

The rate of PTB in the second pregnancy was lower among women with any upward income mobility between births (57.2 per 1000 births) than those who remained in an income Q1 area (61.0 per 1000 births), with a corresponding aRR of 0.95 (95% CI, 0.92 to 0.99) and aARD of −2.9 cases per 1000 births (95% CI, −5.1 to −0.6 per 1000 live births) (eTable 4 in Supplement 1). Again, the associated reduction in the risk of PTB was most pronounced among mothers who changed from a Q1 to Q4 or Q5 income neighborhood between births (eTable 5 in Supplement 1).

### Additional Analysis

There was no significant interaction detected between “neighborhood income mobility between births and immigrant status” and the risk of SMM-M (footnote of eTable 6 in Supplement 1). The respective aRR for upward mobility among immigrants and nonimmigrants are separately shown in eTable 6 in Supplement 1, with each having an inverse association.

For the outcome of SNM-M, there was no significant interaction between “neighborhood income mobility between births and immigrant status” (footnote of eTable 7 in Supplement 1). An inverse association of upward mobility and the risk of SNM-M was only observed among nonimmigrant women (eTable 7 in Supplement 1).

## Discussion

In this large, population-based cohort study of nulliparous Canadian women initially residing in low-income urban areas, changing to any higher residential income neighborhood between first and second births was associated with a lower risk of SMM-M and SNM-M. For SMM-M, this apparent inverse association was consistent for upward movement to any higher-income neighborhood, while for SNM-M, it was most pronounced by a greater degree of upward mobility.

### Other Studies

Few studies have examined neighborhood income mobility in relation to pregnancy outcomes and there is no prior research on SMM-M and SNM-M, which are important public health indicators of maternal and neonatal health and quality of health care.^46,47,48^ The current findings align with prior US research on PTB.^17,18,19,49^ A matched-sibling design in California found that women with upward mobility from a very disadvantaged neighborhood between 2 consecutive live births had a reduced odds of PTB, but not small-for-gestational-age birth weight.^49^ Another study from California observed a lower risk of PTB among White and Latina women with upward income mobility between early life and adulthood, but not in Black women.^19^ African American women who achieved upward mobility from early-life residence in poor Chicago neighborhoods did experience a significant reduction in their risk of PTB, however,^17^ along with a lower risk of small-for-gestational-age birth weight.^18^ As in the current study, the greatest benefit was seen in newborns whose mother had moved to the highest income neighborhoods.^17,18^

Moving from a low- to higher-income neighborhood may be positively associated with maternal health. More affluent neighborhoods tend to have lower crime rates, more safe green spaces, reliable housing, and better access to healthier foods, education, and health services. Such neighborhoods may also introduce fewer chronic stressors, and generally have higher air and water quality, with reduced vehicular traffic.^14,50,51^ Aside from neighborhood factors that may alter the risk of SMM-M, SNM-M, or PTB are those of the woman herself, namely, educational attainment, health literacy, assertiveness, and both mental and physical resilience. Women who tend to move to higher-income areas may possess such attributes, compared with those who remain within a low-income area, and thus, achieve a better health state at the time of their second pregnancy.^52^

A consistent and pronounced association with upward mobility was seen for the outcomes of SMM-M and SNM-M. A cumulative pathway life course model suggests that the longer a person is exposed to socioeconomic challenges, the greater their accumulation of poor physical, psychological and cognitive attributes, which may further affect reproductive health.^53,54^ However, upward social mobility may lessen the accumulation of such adverse exposures,^50,55^ and potentially introduce protective exposures for the mother-to-be. Likewise, a fetus starts off with a 42-week maximum gestational window during which it, too, may be positively influenced by its mother’s place of residence, and a more optimal environment for in utero development.^56^ Future research might evaluate whether downward neighborhood income mobility between pregnancies, contrasted with continued residence in a higher-income area, is associated with adverse birth outcomes.^57,58^

### Public Health and Clinical Relevance

Certainly, greater understanding is needed about the causal pathways that might explain why upward neighborhood income mobility appears to be associated with a lower risk of SMM-M, SNM-M, and PTB. Details should include neighborhood characteristics, particularly local access to high-quality perinatal health care and follow-up care, primary reason for moving, as well as maternal characteristics, behaviors, and experiences that promote or constrain achieving a healthy pregnancy. To overcome some limitations arising from observational study designs, an interventional study using neighborhood-level randomization may be warranted; this can reduce selection bias and unmeasured confounding. Such an approach has been successful in prior research aimed at reducing the risk of obesity and diabetes.^59^ A future study might also investigate whether improving the infrastructure and available services within low-income areas, akin to those found in higher-income neighborhoods, is associated with better pregnancy outcomes for women and infants.^32^ Such a study could include community benefits agreements that aim to prevent mere gentrification, while ensuring that neighborhood improvements are beneficial to the community as a whole, as well as to adjacent areas.^60^

### Strengths and Limitations

This large population-based cohort study was conducted in a province with a high degree of ethnic diversity and immigration, and a universal health care system. Validated data sets captured nearly all hospital births during the study period,^61^ and SMM and SNM outcomes were based on validated measures routinely captured within hospital administrative data. The SMM and SNM composites consist of a broad range of conditions and procedures, which minimizes the risk of under-ascertainment of each outcome.^39^ We also accounted for several important individual-level factors that may confound the relation between neighborhood income mobility and each study outcome, including the interpregnancy birth interval.

Lack of information on early maternal life exposures, race, employment, cigarette or substance use, body mass index, miscarriage, or quality of care could potentially lead to residual confounding. If a woman remained in neighborhood income Q1 during both of her pregnancies, it was not known if she remained within the same domicile. Neighborhood income quartile offers a measure of area- and individual-level income status^62^; however, details of individual-level income were unavailable herein. Women lacking an up-to-date residential address may have been incorrectly assigned a neighborhood income Q; yet, this was unlikely to be the case, as Ontario Health Insurance Plan activity is updated at every antenatal and hospital delivery episode of care. Also, we did not capture if a woman moved more than once between pregnancies in our study, since her residential postal code at the index hospitalization for the first and second deliveries births were used to determine neighborhood income quintile at each birth.

As only singleton births were included herein, the current findings may not apply to women with a multifetal pregnancy, whose risk of SMM and SNM is higher.^63^ Cohort entry also required a woman to have 2 births, which likely introduced selection bias, in that anyone who died after her first birth, underwent a hysterectomy, or who simply did not achieve a second pregnancy would have had a different trajectory after her first index delivery. As only women initially living in low-income urban areas were studied, these findings may not be generalizable to those living in low-income rural areas, where there may be less access to health care services.^64,65^

## Conclusions

In this cohort of universally insured nulliparous Canadian women residing in low-income urban neighborhoods, mobility to a higher-income area between births was associated with a lower risk of SMM-M and SNM-M. Among women living in low-income areas, research is needed to determine whether financial incentives can improve pregnancy outcomes, or whether enhancement of neighborhood factors may reduce adverse maternal and perinatal outcomes.

## Notes

Supplement 1. **eTable 1. **Cohort Entry and Exclusion Criteria, Methods, and Coding to Identify Study Outcomes **eTable 2. **List of ICES Databases Used in the Current Study **eFigure. **Flow Diagram of Cohort Creation **eTable 3. **Rates of Severe Maternal Morbidity or Maternal Mortality (SMM-M) Arising in the Mother’s Birth Hospitalization of the Second Pregnancy, or up to 42 Days Thereafter, and Also of Severe Neonatal Morbidity or Neonatal Mortality (SNM-M) Arising in the Newborn’s Birth Admission of the Second Pregnancy or up to 27 Days Thereafter, Regardless of Neighborhood Income Mobility Between Pregnancies **eTable 4. **Risk of Preterm Birth Between 20^0/7^ to 36^6/7^ Weeks’ Gestation Arising in the Infant’s Birth Admission of the Second Pregnancy, Comparing Infants Whose Mother Moved From a Neighborhood Income Quintile 1 (Q1) to any Higher Neighbourhood Income Quintile (Q2-Q5) Between her First and Second Consecutive Births (any Upward Mobility) vs. Those With no Upward Mobility **eTable 5. **Risk of Preterm Birth Between 20^0/7^ to 36^6/7^ Weeks’ Gestation Arising in the Infant’s Birth Admission of the Second Pregnancy, by Degree of Upward Mobility From a Neighbourhood Income Quintile 1 (Q1) to a Higher Neighborhood Income Quintile (Q2/3 or Q4/5) Between the First and Second Consecutive Birth vs. Those With no Upward Mobility **eTable 6. **Risk of Severe Maternal Morbidity or Maternal Mortality (SMM-M) Arising in the Mother’s Birth Hospitalization of the Second Pregnancy, or up to 42 Days Thereafter, Comparing Women who Moved From a Neighbourhood Income Quintile 1 (Q1) to any Higher Neighborhood Income Quintile (Q2-Q5) Between Their First and Second Consecutive Births (any Upward Mobility) vs. Those With no Upward Mobility, Presented by Maternal Immigrant Status **eTable 7. **Risk of Severe Neonatal Morbidity or Neonatal Mortality (SNM-M) Arising in the Infant’s Birth Admission of the Second Pregnancy, or up to 27 Days Thereafter, Comparing Infants Whose Mother Moved From a Neighborhood Income Quintile 1 (Q1) to any Higher Neighbourhood Income Quintile (Q2-Q5) Between her First and Second Consecutive Births (any Upward Mobility) vs. Those With no Upward Mobility, Presented by Maternal Immigrant Status

Supplement 2.
Data Sharing Statement
