# Errors in Methods, Discussion, and Supplement

**DOI:** 10.1001/jamanetworkopen.2023.23567

**Published:** 2023-06-23

In the Original Investigation titled “Neighborhood Income Mobility and Risk of Neonatal and Maternal Morbidity,”^1^ published May 23, 2023, neighborhood income quintile was incorrectly referred to as neighborhood income quartile in the Methods and Discussion sections. In addition, the Supplement cited reference 42 in eTable 4, eTable 5, eTable 6, and eTable 7, when it should have cited reference 41. This article has been corrected.^1^
