# Supplementary material for: Neighborhood Income Mobility and Risk of Neonatal and Maternal Morbidity
Source: JAMA Netw Open. 2023 May 23;6(5):e2315301. doi: 10.1001/jamanetworkopen.2023.15301 (PMC10208146; doi:10.1001/jamanetworkopen.2023.15301)
Supplement: Supplement 1. — eTable 1. Cohort Entry and Exclusion Criteria, Methods, and Coding to Identify Study Outcomes eTable 2. List of ICES Databases Used in the Current Study eFigure. Flow Diagram of Cohort Creation eTable 3. Rates of Severe Maternal Morbidity or Maternal Mortality (SMM-M) Arising in the Mother’s Birth Hospitalization of the Second Pregnancy, or up to 42 Days Thereafter, and Also of Severe Neonatal Morbidity or Neonatal Mortality (SNM-M) Arising in the Newborn’s Birth Admission of the Second Pregnancy or up to 27 Days Thereafter, Regardless of Neighborhood Income Mobility Between Pregnancies eTable 4. Risk of Preterm Birth Between 200/7 to 366/7 Weeks’ Gestation Arising in the Infant’s Birth Admission of the Second Pregnancy, Comparing Infants Whose Mother Moved From a Neighborhood Income Quintile 1 (Q1) to any Higher Neighbourhood Income Quintile (Q2-Q5) Between her First and Second Consecutive Births (any Upward Mobility) vs. Those With no Upward Mobility eTable 5. Risk of Preterm Birth Between 200/7 to 366/7 Weeks’ Gestation Arising in the Infant’s Birth Admission of the Second Pregnancy, by Degree of Upward Mobility From a Neighbourhood Income Quintile 1 (Q1) to a Higher Neighborhood Income Quintile (Q2/3 or Q4/5) Between the First and Second Consecutive Birth vs. Those With no Upward Mobility eTable 6. Risk of Severe Maternal Morbidity or Maternal Mortality (SMM-M) Arising in the Mother’s Birth Hospitalization of the Second Pregnancy, or up to 42 Days Thereafter, Comparing Women who Moved From a Neighbourhood Income Quintile 1 (Q1) to any Higher Neighborhood Income Quintile (Q2-Q5) Between Their First and Second Consecutive Births (any Upward Mobility) vs. Those With no Upward Mobility, Presented by Maternal Immigrant Status eTable 7. Risk of Severe Neonatal Morbidity or Neonatal Mortality (SNM-M) Arising in the Infant’s Birth Admission of the Second Pregnancy, or up to 27 Days Thereafter, Comparing Infants Whose Mother Moved From a Neighborhood Income Quintile 1 (Q [file jamanetwopen-e2315301-s001.pdf]

Supplemental Online Content

Jairam JA, Vigod SN, Siddiqi A, et al. Neighborhood income mobility and risk of neonatal and maternal morbidity. *JAMA Netw Open*. 2023;6(5):e2315301. doi:10.1001/jamanetworkopen.2023.15301

eTable 1. Cohort Entry and Exclusion Criteria, Methods, and Coding to Identify Study Outcomes

eTable 2. List of ICES Databases Used in the Current Study

eFigure. Flow Diagram of Cohort Creation

eTable 3. Rates of Severe Maternal Morbidity or Maternal Mortality (SMM-M) Arising in the Mother's Birth Hospitalization of the Second Pregnancy, or up to 42 Days Thereafter, and Also of Severe Neonatal Morbidity or Neonatal Mortality (SNM-M) Arising in the Newborn's Birth Admission of the Second Pregnancy or up to 27 Days Thereafter, Regardless of Neighborhood Income Mobility Between Pregnancies

eTable 4. Risk of Preterm Birth Between $20^{0/7}$ to $36^{6/7}$ Weeks' Gestation Arising in the Infant's Birth Admission of the Second Pregnancy, Comparing Infants Whose Mother Moved From a Neighborhood Income Quintile 1 (Q1) to any Higher Neighbourhood Income Quintile (Q2-Q5) Between her First and Second Consecutive Births (any Upward Mobility) vs. Those With no Upward Mobility

eTable 5. Risk of Preterm Birth Between $20^{0/7}$ to $36^{6/7}$ Weeks' Gestation Arising in the Infant's Birth Admission of the Second Pregnancy, by Degree of Upward Mobility From a Neighbourhood Income Quintile 1 (Q1) to a Higher Neighborhood Income Quintile (Q2/3 or Q4/5) Between the First and Second Consecutive Birth vs. Those With no Upward Mobility

eTable 6. Risk of Severe Maternal Morbidity or Maternal Mortality (SMM-M) Arising in the Mother's Birth Hospitalization of the Second Pregnancy, or up to 42 Days Thereafter, Comparing Women who Moved From a Neighbourhood Income Quintile 1 (Q1) to any Higher Neighborhood Income Quintile (Q2-Q5) Between Their First and Second Consecutive Births (any Upward Mobility) vs. Those With no Upward Mobility, Presented by Maternal Immigrant Status

eTable 7. Risk of Severe Neonatal Morbidity or Neonatal Mortality (SNM-M) Arising in the Infant's Birth Admission of the Second Pregnancy, or up to 27 Days Thereafter, Comparing Infants Whose Mother Moved From a Neighborhood Income Quintile 1 (Q1) to any Higher Neighbourhood Income Quintile (Q2-Q5) Between her First and Second Consecutive Births (any Upward Mobility) vs. Those With no Upward Mobility, Presented by Maternal Immigrant Status

This supplemental material has been provided by the authors to give readers additional information about their work.

© 2023 Jairam JA et al. *JAMA Network Open.*

eTable 1. Cohort Entry and Exclusion Criteria, Methods, and Coding to Identify Study Outcomes

Assessment	Timing	Disease, procedure or condition	ICD-10-CA or CCI codes in CIHI-DAD	Diagnostic & fee codes in OHIP	Other sources
Inclusion criteria	April 1, 2002 to December 31, 2019, at the time of the mother's index delivery hospitalization date, for the first and second pregnancies	• All hospital singleton livebirths and stillbirths at $20^{0/7}$ to $42^{0/7}$ weeks' gestation among nulliparous women who were initially residing in a lowest income Q1 urban neighbourhood in Ontario at the time of their first birth, and who had a second consecutive birth also in Ontario.<br>• Births limited to women aged 15-50 years old with a valid OHIP/IKN number from MOMBABY for the first and second index birth hospitalization for delivery.<br>• Stillbirth: newborn with no signs of life at birth or *in utero*, and ≥ $20^{0/7}$ weeks' gestation and ≥ 250 g at birth.	MOMBABY (links the hospital admission records of delivering mothers and newborns in CIHI-DAD)	--	IRCC-PRD, RPDB
	Same as above	• Mother's postal code was used to derive neighbourhood income Q.	--	--	RPDB, PCCF+, Statistic Canada census data
Exclusion criteria	At the mother's index delivery hospitalization, for the first pregnancy	Women with a previous birth (not nulliparous)	MOMBABY	--	--
	Same	Women without a second birth during study period	MOMBABY		
	Same	Women living in income quintile 2 to 5	--	--	RPDB, PCCF+, Statistic Canada census data
	At the infant's index birth admission, for the first and second pregnancies	Infant (livebirths & stillbirths) birthweight <250 g or missing	MOMBABY:<br>a. B_WEIGHT < 250<br>b. B_WEIGHT not in MOMBABY	--	--
	Same	Infant gestational age at birth is missing	MOMBABY:<br>a. B_GESTWKS_DEL not in MOMBABY<br>b. gestational age <20 or ≥43 weeks	--	--
	Same	Multiple births	MOMBABY (M_MULTIBIRTH='T' or B_MULTIBIRTH='T')	--	--

© 2023 Jairam JA et al. *JAMA Network Open.*

Assessment	Timing	Disease, procedure or condition	ICD-10-CA or CCI codes in CIHI-DAD	Diagnostic & fee codes in OHIP	Other sources
	At the mother's index delivery hospitalization, for the first and second pregnancies	Women <15 or >50 years or age missing	--	--	RPDB: M_IKN age < 15 or > 50 or missing
	Same	Women who were a non-Ontario resident	--	--	RPDB: M_IKN non-Ontario resident (substr (prcddablk, 1,2 ne '35')
	Same	First birth - Women who had an invalid OHIP number or hospital number<br><br>Second birth - Women who had an invalid OHIP number or hospital number & infants who had an invalid OHIP number	First birth - MOMBABY/RPDB: Invalid M_IKN (maternal IKN)<br>a. VALIKN ne 'V'<br>b. M_IKN not in RPDB (according to no sex and no bdate)<br>c. M_IKN with sex ='M' in RPDB<br><br>Second birth- MOMBABY/RPDB: Invalid M_IKN or B_IKN<br>a. VALIKN ne 'V'<br>b. M_IKN not in RPDB (according to no sex & no bdate)<br>c. M_IKN with sex ='M' in RPDB	--	--
	Same	Women ineligible for OHIP	--	--	RPDB
	Same	Women with income quintile missing	--	--	RPDB, PCCF+, Statistics Canada census data
	Same	First birth – Records with warning for mother's IKN or KEY<br><br>Second birth- Records with warning for mother's or infants' IKN or KEY	MOMBABY/RPBD: warning for IKN/KEY (WARN not ="N"(No Warning)). Include N=no warning	--	--
	Same	Living in non-urban areas at delivery or missing	--	--	RPDB: (rural='0')
	1 to 365 days before the mother's index delivery hospitalization, for the second pregnancy	Women ineligible for OHIP	--	--	RPDB

© 2023 Jairam JA et al. *JAMA Network Open.*

Assessment	Timing	Disease, procedure or condition	ICD-10-CA or CCI codes in CIHI-DAD	Diagnostic & fee codes in OHIP	Other sources
	At the mother's index delivery hospitalization, for the second pregnancy	< 161 days between infant birth hospitalization dates for the first and second consecutive births (e.g., infant birth dates)	MOMBABY: B_BDATE	--	--
	At the time of arrival to Canada	Refugees, other immigrants	--	--	IRCC-PRD
	Same	Non-refugee immigrants with a landing date prior to their birth date, or after their infant's birth date, or missing	RPDB	--	IRCC-PRD
	Same	Canadian-born women who are also classified as an immigrant, or women missing country of birth	--	--	IRCC-PRD
Main study exposure	At the mother's index delivery hospitalization, for the **first** and **second** pregnancies	Neighbourhood income mobility defined as:<br><br>***Any upward mobility –*** moving from an urban neighbourhood income Q1 area to any higher urban neighbourhood income Q2-Q5 area between the first and second consecutive births<br><br>***No upward mobility*** - (referent) remaining in an urban income Q1 neighbourhood between the first and second consecutive births	--	--	RPDB: PSTLCODE<br>Statistics Canada PCCF+<br>census data
Secondary study exposure	Same	The degree of neighbourhood income mobility between the first and second consecutive births, categorized into 4 income mobility patterns - moving from an urban income Q1 neighbourhood to a: i) Q2 neighbourhood, ii) Q3 neighbourhood, iii) Q4/5 neighbourhood, or iv) no upward mobility (remaining in an urban income Q1 neighbourhood between the first and second consecutive births) (referent)	--	--	RPDB: PSTLCODE<br>Statistics Canada PCCF+<br>census data

© 2023 Jairam JA et al. *JAMA Network Open.*

Main Study outcomes	Between 0 days (at the mother's index delivery hospitalization, for the second pregnancy) and up to 42 days thereafter - including livebirths and stillbirths	(1) Severe maternal morbidity (SMM)	Severe preeclampsia and (HELLP) syndrome:<br>O14.1, or O14.2<br><br>Eclampsia:<br>O15<br><br>Cerebral venous thrombosis in pregnancy, or in the puerperium:<br>O22.5, or O87.3<br><br>Acute fatty liver with red blood cell (RBC) transfusion or plasma transfusion:<br>O26.6 + (CIHI BTREDBC = 1 or CIHI BTPLASMA = 1)<br><br>Pulmonary, cardiac, and CNS complications of anesthesia during pregnancy, the puerperium or labour and delivery:<br>O29.0, O29.1, O29.2, O89.0, O89.1, O89.2, O74.0, O74.1, O74.2 or O74.3<br><br>Placenta previa with hemorrhage with RBC transfusion:<br>O44.1 + CIHI BTREDBC = 1<br><br>Placental abruption with coagulation defect:<br>O45.0<br><br>Antepartum hemorrhage with coagulation defect:<br>O46.0<br><br>Intrapartum hemorrhage with coagulation defect:<br>O67.0	--	--

© 2023 Jairam JA et al. *JAMA Network Open.*

			Intrapartum hemorrhage with RBC transfusion:<br>O67 + CIHI BTREDBC = 1<br><br>Rupture of the uterus with RBC transfusion, procedures to the uterus or hysterectomy:<br>(O71.0 or O71.1) + any of the following:<br>• CIHI BTREDBC = 1, or<br>• (1.RM.13, 1.KT.51, 5.PC.91.LA or 5.PC.91.HV) + CIHI BTREDBC = 1, or<br>• (5.MD.60.RC, 5.MD.60.RD, 5.MD.60.KE, 5.MD.60.CB or 1.RM.89.LA[a]), or<br>• 1.RM.87.LA-GX<br><br>[a]**NOTE**: **1.RM.89.LA** is included only if codes 1.PL.74, 1.RS.74 or 1.RS.80 are NOT also present<br><br>Postpartum hemorrhage with RBC transfusion, procedures to the uterus or hysterectomy:<br>O72 + any of the following:<br>• BTREDBC = 1, or<br>• (1.RM.13, 1.KT.51, 5.PC.91.LA or 5.PC.91.HV) + BTREDBC = 1, or<br>• (5.MD.60.RC, 5.MD.60.RD, 5.MD.60.KE, 5.MD.60.CB or 1.RM.89.LA[b]), or<br>• 1.RM.87.LA-GX<br><br>[b]**NOTE**: **1.RM.89.LA** is included only if codes 1.PL.74, 1.RS.74 or 1.RS.80 are NOT also present	--	--

© 2023 Jairam JA et al. *JAMA Network Open.*

Assessment	Timing	Disease, procedure or condition	ICD-10-CA or CCI codes in CIHI-DAD	Diagnostic & fee codes in OHIP	Other sources
			Cardiac conditions:<br>O74.2, O89.1, O90.3, I21, I22, I42, I43, I46, I49.0, I50, J81, 1.HZ.09 or 1.HZ.30<br><br>Obstetric shock:<br>O75.1, R57, T80.5 or T88.6<br><br>Septicemia during labour:<br>O75.3<br><br>Complications of obstetric surgery and procedures:<br>O75.4<br><br>Puerperal sepsis:<br>O85<br><br>Obstetric embolism:<br>O88<br><br>Acute renal failure:<br>O90.4, N17, N19 or N99.0<br><br>Disseminated intravascular coagulation:<br>D65<br><br>Sickle cell anemia with crisis:<br>D57.0<br><br>Acute psychosis:<br>F53.1 or F23<br><br>Status epilepticus:<br>G41	--	--

© 2023 Jairam JA et al. *JAMA Network Open.*

			Cerebral edema or coma:<br>G93.6 or R40.2<br><br>Cerebrovascular diseases: subarachnoid and intracranial hemorrhage, cerebral infarction, stroke:<br>I60, I61, I62, I63 or I64<br><br>Status asthmaticus:<br>J45.01, J45.11, J45.81 or J45.91<br><br>Adult respiratory distress syndrome:<br>J80<br><br>Acute abdomen:<br>K35, K37, K65, N73.3 or N73.5<br><br>Hepatic failure:<br>K71 or K72<br><br>Assisted ventilation through endotracheal tube:<br>1.GZ.31.CA-ND<br><br>Assisted ventilation through tracheostomy:<br>1.GZ.31.CR-ND<br><br>Hysterectomy:<br>5.MD.60.RC, 5.MD.60.RD, 5.MD.60.KE, 5.MD.60.CB, 1.RM.89.LA (exclude if 1.PL.74, 1.RS.74 or 1.RS.80 code also present), 1.RM.87.LA-GX<br><br>Dialysis:<br>1.PZ.21	--	--

© 2023 Jairam JA et al. *JAMA Network Open.*

Assessment	Timing	Disease, procedure or condition	ICD-10-CA or CCI codes in CIHI-DAD	Diagnostic & fee codes in OHIP	Other sources
			Evacuation of incisional hematoma with RBC transfusion:<br>5.PC.73.JS + CIHI BTREDBC = 1<br><br>Repair of bladder, urethra, or intestine:<br>5.PC.80.JR, 1.NK.80, 1.NM.80<br><br>Procedures to the uterus/pelvic vessels with RBC transfusion:<br>(1.RM.13, 1.KT.51, 5.PC.91.LA, 5.PC.91.HV) + CIHI BTREDBC = 1<br><br>Surgical or manual correction of inverted uterus for vaginal births only:<br>5.PC.91.HQ or 5.PC.91.HP, restricted to vaginal births (i.e., absence of caesarean 5.MD.60)<br><br>Reclosure of caesarean wound with RBC transfusion:<br>(5.PC.80.JM, 5.PC.80.JH) + CIHI BTREDBC = 1<br><br>Curettage with RBC transfusion:<br>(5.PC.91.GA, 5.PC.91.GC, 5.PC.91.GD) + CIHI BTREDBC = 1<br><br>Maternal ICU admission:<br>SCU in (‘10’, ’20’, ’25’, ’30’, ’35’, ’40’,’45’,’60’,’80’)	--	

© 2023 Jairam JA et al. *JAMA Network Open.*

Assessment	Timing	Disease, procedure or condition	ICD-10-CA or CCI codes in CIHI-DAD	Diagnostic & fee codes in OHIP	Other sources
	Between 0 days (at the mother's index delivery hospitalization, for the second pregnancy) and up to 42 days thereafter - including livebirths and stillbirths	SMM or all-cause maternal mortality	See above for SMM indicators, and O96 or other all-cause mortality (CIHI-DAD)	--	RPDB, ORGD

© 2023 Jairam JA et al. *JAMA Network Open.*

	Between 0 days (at the infant's birth hospitalization, for the second pregnancy) and up to 27 days thereafter - including livebirths only	(3) Severe neonatal morbidity (SNM)	Gestational age < 32 weeks<br>MOMBABY: B_GESTWKS_DEL<br><br>Birthweight < 1500 grams<br>MOMBABY: B_WEIGHT<br><br>Respiratory distress syndrome<br>P22.0<br><br>Seizures<br>P90, R56<br><br>Cerebral infarction<br>I63<br><br>Intraventricular haemorrhage (grades 3 and 4)<br>P52.2<br><br>Periventricular leukomalacia<br>P91.2<br><br>Birth Trauma (intracranial hemorrhage paralysis due to brachial plexus injury, skull or long bone fracture)<br>P10.0-3, P13.0, P13.2-3, P14.0-1<br><br>Hypoxic ischemic encephalopathy<br>P91.5, P91.8, P91.6<br><br>Necrotising enterocolitis<br>P77<br><br>Sepsis/septicaemia (streptococcus staphylococcus, E.coli, unspecified gram negative)<br>P36.0-8; B95.1; B96.2<br><br>Pneumonia<br>P23; J12-18	Hypothermia (therapeutic) induction<br>G210	--

© 2023 Jairam JA et al. *JAMA Network Open.*

			Other respiratory (primary atelectasis, respiratory failure)<br>P28.0; P28.5<br><br>Chronic respiratory disease originating in the perinatal period<br>P27<br><br>Bacterial meningitis<br>G00-03; G05<br><br>Resuscitation<br>1HZ30JN, 1HZ30JY, 1GZ30CJ, 1GZ30CJNB, 1GZ30JH<br><br>Ventilatory support (mechanical ventilation and/or CPAP)<br>1GZ31CAEP, 1GZ31CAND, 1GZ31CAPK, 1GZ31CBND, 1GZ31CRND, 1GZ31GPND, 1GZ31JAGX, 1GZ31JAMD, 1GZ31JANC, 1GZ31JAPK<br><br>Central venous or arterial catheter<br>1KV53HACH, 1KV53HAFT, 1KV53LAFT, 2IM28GP, 2LZ28GQPL, 2LZ28GRPL, 2LZ28JAPL, 1KX53HACH, 1KX53HAFT, 1KX53 LAFT, 2LZ28GQPL, 2LZ28GRPL<br><br>Pneumothorax requiring intercostal catheter<br>1GV52DA, 1GV52DATS, 1GV52HA, 1GV52HAHE, 1GV52HATK, 1GV52LA, 1GV52LATS, 1GV52LAXXE, 1GV54JATS, 1GV55JATS<br>P25.1		--

© 2023 Jairam JA et al. *JAMA Network Open.*

Assessment	Timing	Disease, procedure or condition	ICD-10-CA or CCI codes in CIHI-DAD	Diagnostic & fee codes in OHIP	Other sources
			Any intravenous fluids<br>1LZ35CAE6, 1LZ35HAC1, 1LZ35HAC5,<br>1LZ35HAC6, 1LZ35HAC7, 1LZ35HAE6,<br>1LZ35HAT7, 1LZ35HAT9, 1LZ35HAZ9, 1LZ35HHC1, 1LZ35HHC5, 1LZ35HHC6, 1LZ35HHC7, 1LZ35HHE0, 1LZ35HHE6, 1LZ35HHT7, 1LZ35HHT9, 1LZ35HHZ9, 1LZ35HRC5, 1LZ35HRC6, 1LZ35HRC7, 1LZ35HRT9, 1LZ35HRZ9		--

© 2023 Jairam JA et al. *JAMA Network Open.*

Assessment	Timing	Disease, procedure or condition	ICD-10-CA or CCI codes in CIHI-DAD	Diagnostic & fee codes in OHIP	Other sources
			<u>Any body cavity surgical procedure</u><br>1AA52, 1AA87, 1AC87, 1AE87, 1AF87, 1AG87, 1AJ87, 1AK87, 1AN52, 1AN59, 1AN87, 1AP59, 1AP72, 1AP87, 1AW59, 1AW72, 1AW87, 1AX87, 1BA72, 1BA80, 1BA87, 1BB72, 1BB80, 1BB87, 1BD72, 1BD80, 1BD87, 1BF80, 1BG72, 1BG80, 1BG87, 1BK59, 1BM72, 1BM80, 1BM87, 1BN72, 1BN80, 1BN87, 1BP72, 1BP80, 1BP87, 1BQ72, 1BQ80, 1BQ87, 1BS72, 1BS80, 1BS87, 1BT72, 1BT80, 1BT87, 1GA87, 1GA89, 1GB87, 1GB89, 1GD89, 1GE80, 1GE87, 1GE89, 1GE91, 1GH84, 1GJ86, 1GJ87, 1GK87, 1GK89, 1GM80, 1GM86, 1GM87, 1GN92, 1GR87, 1GR89, 1GR91, 1GT78, 1GT87, 1GT89, 1GT91, 1GV87, 1GV89, 1GW87, 1GX80, 1GX86, 1GX87, 1GY70, 1GY72, 1GY86, 1HJ76, 1HJ82, 1HN87, 1HP76, 1HP78, 1HP80, 1HP82, 1HP83, HP87, 1HR80, 1HR84, 1HR87, 1HS80 (excl. 1HS80G), 1HS90, 1HT80 (excl. 1HT80G), 1HT89, 1HT90, 1HU80 (excl. 1HU80G), 1HU90, 1HV80 (excl. 1HV80G), 1HV90, 1HW78, 1HW79, 1HX80, 1HX87, 1HX80, 1HZ87, 1IA76, 1IA80, 1IA87, 1IB76, 1IB79, 1IB80, 1IB82, 1IB87, 1IC76, 1IC80, 1IC82, 1IC87, 1ID76, 1ID80, 1ID82,		--

© 2023 Jairam JA et al. *JAMA Network Open.*

Assessment	Timing	Disease, procedure or condition	ICD-10-CA or CCI codes in CIHI-DAD	Diagnostic & fee codes in OHIP	Other sources
			1ID86, 1ID87, 1IF83, 1IJ76, 1IJ80, 1IM76, 1IM80, 1IM82, 1IM83, 1IM87, 1IN83, 1IN84, 1IN87, 1JE57 (excl. 1JE57G), 1JE76, 1JE80, 1JE87, 1JJ76, 1JJ80, 1JK76, 1JK80, 1JK87, 1JW51 (excl. 1JW51G), 1JW57, 1JW76, 1LA84, 1LC84, 1LD84, 1NA72, 1NA74, 1NA76, 1NA77, 1NA80, 1NA84, 1NA86, 1NA87, 1NA88, 1NA89, 1NA90, 1NA91, 1NA92, 1NE80, 1NF76, 1NF78, 1NF80, 1NF82, 1NF84, 1NF86, 1NF87 (excl. 1NF87B), 1NF89, 1NF90, 1NF91, 1NF92, 1NK76, 1NK77, 1NK80, 1NK82, 1NK84, 1NK87 (excl. 1NK87B), 1NM74, 1NM76, 1NM77, 1NM80, 1NM82, 1NM87 (excl. 1NM87B), 1NM89, 1NM91, 1NP72, 1NP73, 1NP86, 1NQ74 (excl. 1NQ74B), 1NQ80, 1NQ84, 1NQ86, 1NQ87 (excl. 1NQ87B), 1NQ89, 1NQ90, 1NT80, 1NT84, 1NT86, 1NT87, 1NV89, 1OA87, 1OB87, 1OB89, 1OD76, 1OD89, 1OE76, 1OE80, 1OE89, 1OJ76 (excl. 1OJ76B), 1OJ87, 1OJ89, 1OK87, 1OK89, 1OK91, 1OT72, 1OT87, 1OT91, 1PB87, 1PB89, 1PC80, 1PC87 (excl. 1PC87D), 1PC89, 1PC91, 1PE57 (excl. 1PE57BD), 1PE80 (excl. 1PE80D), 1PE82, 1PE87 (excl. 1PE87D), 1PE89 (excl. 1PE89D), 1PG76, 1PG77, 1PG80 (excl.1PG80D), 1PG86, 1PG89, 1PL74 (excl.1PL74CD), 1PL80, 1PM79, 1PM86, 1PM87 (excl. 1PM87B),		--

© 2023 Jairam JA et al. *JAMA Network Open.*

Assessment	Timing	Disease, procedure or condition	ICD-10-CA or CCI codes in CIHI-DAD	Diagnostic & fee codes in OHIP	Other sources
			1PM89, 1PM90, 1PM91, 1PM92, 1QE53, 1QE80, 1QE82, 1QE84, 1QE87, 1QE89, 1QG89, 1QM74, 1QM80, 1QM87, 1QM89, 1QM91, 1QN82, 1QT87, 1QT91, 1RB74, 1RB80, 1RB83, 1RB87, 1RB89, 1RD89, 1RF51, 1RF72, 1RF74, 1RF80, 1RF87, 1RF89, 1RM87 (excl.1RM87B), 1RM89, 1RM91, 1RN87, 1RN89, 1RS74, 1RS80, 1RS86, 1RS87, 1RS89, 1RW87, 1RW88, 1RW91, 1RW92, 1SA74, 1SA75, 1SA80, 1SA89, 1SC74, 1SC75, 1SC80, 1SC87, 1SC89, 1SE53, 1SE89 (excl. 1SE89D), 1SF80, 1SF87, 1SF89, 1SG80, 1SG87, 1SH87, 1SM74, 1SM80, 1SM87, 1SN87, 1SN93, 1SQ74, 1SQ80, 1SQ87, 1SQ91, 1SQ93, 1SW74, 1SY80, 1SY84, 1SY87, 1SZ87, 1VA53, 1VA74, 1VA75, 1VA80, 1VA87, 1VA93, 1VC74, 1VC80, 1VC87, 1VC91, 1VC93, 1VE80, 1VG53, 1VG55, 1VG72, 1VG73, 1VG74, 1VG75, 1VG80, 1VG87, 1VG93, 1VK80, 1VK87, 1VK89, 1VL80, 1VL87, 1VM80, 1VM87, 1VN80, 1VN87, 1VP74, 1VP80, 1VP87, 1VP89, 1VQ74, 1VQ79, 1VQ80, 1VQ82, 1VQ87, 1VQ91, 1VQ93, 1VS72, 1VS80, 1VX87		--
	Between 0 days (at the infant's index birth admission, for the second pregnancy) and up to 27 days thereafter - including livebirths only	SNM or all-cause neonatal mortality	See above for SNM indicators and O96 or other all-cause mortality	--	RPDB, ORGD

© 2023 Jairam JA et al. *JAMA Network Open.*

Assessment	Timing	Disease, procedure or condition	ICD-10-CA or CCI codes in CIHI-DAD	Diagnostic & fee codes in OHIP	Other sources
	At the infant's index birth admission for the second pregnancy – including livebirths and stillbirths	(4) Preterm (PTB): the birth of a liveborn or stillborn infant between 20 $^{0/7}$ to 36 $^{6/7}$ weeks' gestation	MOMBABY: B_GESTWKS_DEL between 20 $^{0/7}$ to 36 $^{6/7}$ completed weeks	--	--
Covariates (for all 3 main outcomes)	At the mother's index delivery hospitalization, for the second pregnancy	Maternal age	MOMBABY: M_AGE	--	--
	At the infant's index birth admission, for first and the second pregnancies	Birth interval: Time (months) elapsed between infant's index birth admission, for first and the second pregnancies.<br>Calculated by subtracting the infant's index birth admission date for the first pregnancy from the infant's index birth admission date for the second pregnancy. Categories (months): <18, 18-36, 37-60, 61-119, ≥120	MOMBABY: B_BDATE	--	--
	At the time of the mother's arrival to Canada	World Region of Birth: Canada, Western Nations & Europe, Hispanic America, Caribbean, Sub-Saharan Africa, Middle East & North Africa, East Asia & Pacific, South Asia	--	--	IRCC-PRD: COUNTRY_BIRTH
	Reported 1 to 365 days before the mother's index delivery hospitalization, for the second pregnancy	Pre-pregnancy hypertension: elevated blood pressure, a systolic blood pressure > 140 mmHg and/or a diastolic blood pressure > 90 mmHg	401, 405, 642.0-642.2, 642.7 [I10, I15, O10, O11]	401	HYPER
	i) Reported 1 to 365 days before the mother's index delivery hospitalization, for the second pregnancy<br>ii) At the mother's index delivery hospitalization, for the second pregnancy	Composite - of the following 2 variables:<br>i) Pre-pregnancy diabetes mellitus: high levels of glucose in the blood.<br><br>ii) Gestational diabetes: any degree of glucose intolerance with onset/first diagnosis during pregnancy	250, 648.8 [E10, E11, E13, E14, O244]<br><br>O24	250<br><br>--	ODD<br><br>--
Covariates (specific to the outcome of SNM-M)	At the infant's index birth admission for the second pregnancy	Congenital anomaly	ICD-10 Q00-Q89 (also requiring the exclusion of Q90-Q99)	--	--

Abbreviations: CIHI: Canadian Institute for Health Information; DAD: CCI: Canadian Classification of Health Interventions; Discharge Abstract Database; ICD-10-CA: International Classification of Diseases, 10th Revision, Canada; HYPER: Ontario Hypertension Dataset; IRCC-PRD: Immigration, Refugees, and Citizenship Canada Permanent Resident Database; ODD: Ontario Diabetes Dataset; OHIP: Ontario Health Insurance Plan; ORG-D: Office of the Registrar General Deaths; PCCF+: Postal code conversion file plus (Statistics Canada); RPDB: Registered Persons Database.

© 2023 Jairam JA et al. *JAMA Network Open.*

eTable 2. List of ICES Databases Used in the Current Study

Dataset name	Description
Canadian Institute for Health Information Discharge Abstract Database (CIHI-DAD)	Captures all in-patient hospital admission records including obstetric deliveries and deaths. Diagnostic codes are based on the *International Statistical Classification of Diseases and Related Health Problems, Tenth Revision, Canada* (*ICD-10-CA*), and procedural codes are based on the *Canadian Classification of Health Interventions (CCI)*.
Immigration, Refugees and Citizenship Canada Permanent Residents Database (IRCC-PRD)	Contains demographic information on all international migrants who obtained permanent residency in Canada from January 1985 to May 2017.
Linked Delivering Mothers and Newborns (MOMBABY)	Derived from CIHI-DAD, provides linked inpatient hospital admission records of mothers and their infants.
Office of the Registrar General - Deaths (ORG-D)	An annual dataset including the date and cause of death, for all deaths registered in Ontario (1990-2021).
Ontario Diabetes Dataset (ODD)	A dataset containing all the people in Ontario diagnosed with diabetes.
Ontario Hypertension Dataset (HYPER)	A dataset containing all the people in Ontario diagnosed as having hypertension.
Ontario Health Insurance Plan Claims Database (OHIP)	Contains information about inpatient and ambulatory visits, consultations and procedures provided to Ontario residents eligible for Ontario's publicly funded health insurance system by fee-for-service health care practitioners (e.g., physicians, optometrists, laboratories for diagnostic tests etc.).
Postal Code Conversion File Plus (PCCF+)	A digital file that links the Canada Post Corporation (CPC) six-character postal code and Statistics Canada's standard geographic areas (e.g., dissemination area). Area-level income quintiles ranges from Q1 (lowest) to Q5 (highest) income neighbourhoods.
Registered Persons Database (RPDB)	Includes vital status and sociodemographic information about all individuals who have ever received an Ontario Health Insurance Plan (OHIP) number (e.g., date of birth, sex, and postal code).
Statistic Canada Census	Information from the Canadian Census, statistical information about the population including population counts and various levels of geography (e.g., census metropolitan areas, communities, census tracts etc.)

© 2023 Jairam JA et al. *JAMA Network Open.*

eFigure 1. Flow Diagram of Cohort Creation

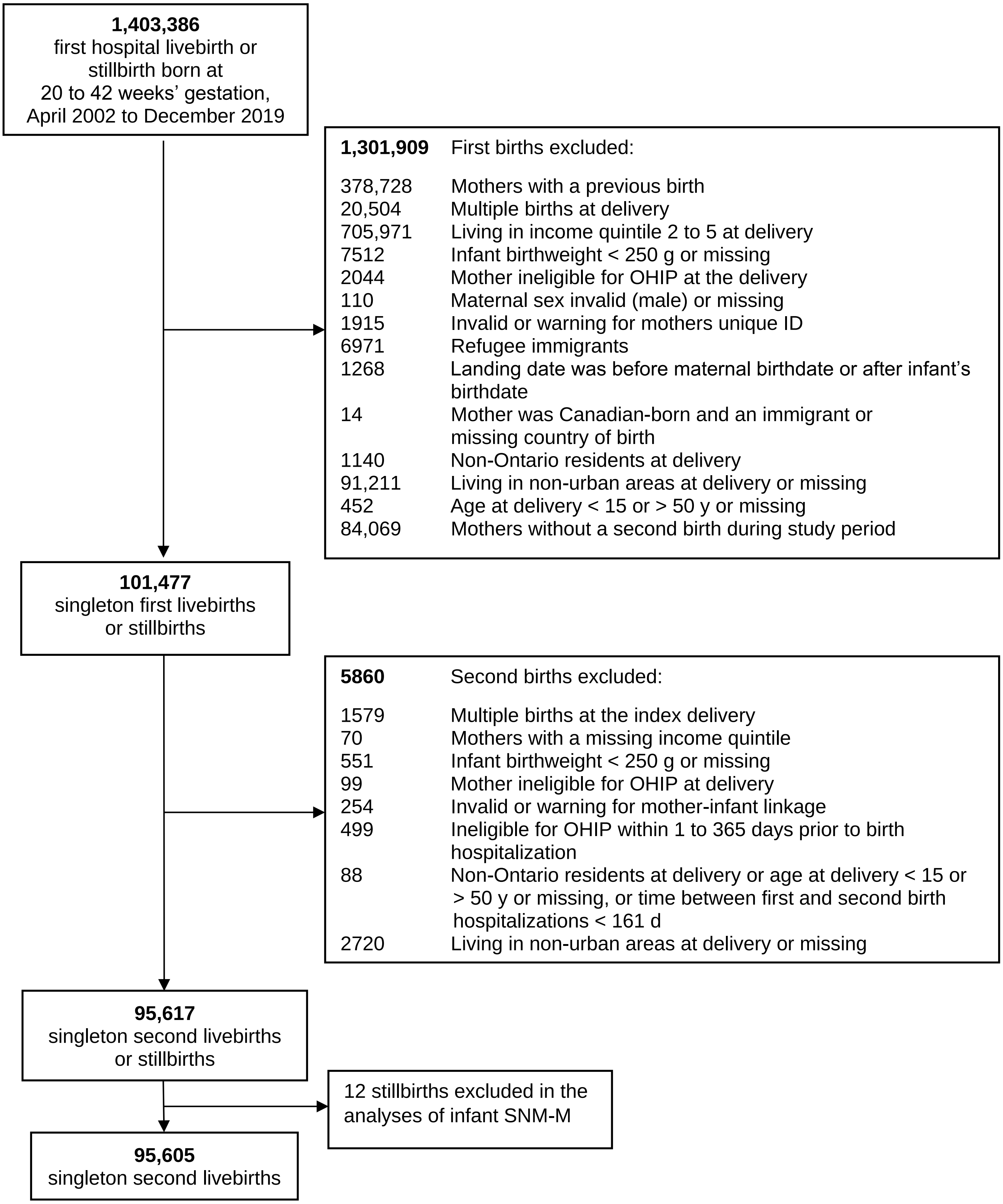

© 2023 Jairam JA et al. *JAMA Network Open.*

eTable 3. Rates of Severe Maternal Morbidity or Maternal Mortality (SMM-M) Arising in the Mother's Birth Hospitalization of the Second Pregnancy, or up to 42 Days Thereafter, and Also of Severe Neonatal Morbidity or Neonatal Mortality (SNM-M) Arising in the Newborn's Birth Admission of the Second Pregnancy or up to 27 Days Thereafter, Regardless of Neighborhood Income Mobility Between Pregnancies

Also presented are the top 20 most common SMM and SNM indicators, respectively. Data are limited to nulliparous women who were initially residing in a lowest income Q1 neighbourhood at their first birth, and who had a second birth also in Ontario. SMM-M rates include all singleton hospital livebirths or stillbirths at $20^{0/7}$ to $42^{0/7}$ weeks' gestation. SNM-M rates include all singleton hospital livebirths at $20^{0/7}$ to $42^{0/7}$ weeks' gestation.

**Mothers in the second pregnancy (N = 95,617)**	**No. of events**	**Rate per 1000 births**		**Newborns in the second pregnancy (N = 95,605)**	**No. of events**	**Rate per 1000 livebirths**
SMM or maternal mortality	1218	12.7		***SNM or neonatal mortality***	4742	49.6
Maternal mortality, with or without concomitant SMM	9	0.09		***Neonatal mortality, with or without concomitant SNM***	212	2.2
Top 20 SMM indicators				***Top 20 SNM indicators***		
Postpartum hemorrhage with red cell transfusion, or procedures to the uterus or hysterectomy	307	3.2		Ventilatory support	3050	31.9
ICU admission	244	2.6		Any intravenous fluids	1550	16.2
Puerperal sepsis	215	2.2		Respiratory distress syndrome	1015	10.6
Severe preeclampsia & HELLP syndrome	137	1.4		Gestational age < 32 weeks	752	7.9
Hysterectomy	119	1.2		Other respiratory (primary atelectasis, respiratory failure)	709	7.4
Curettage with red cell transfusion	78	0.8		Birthweight < 1500 g	661	6.9
Repair of bladder, urethra, or intestine	76	0.8		Sepsis/septicaemia	311	3.3
Complications of obstetric surgery & procedures	74	0.8		Central venous or arterial catheter	298	3.1
Cardiac conditions	71	0.7		Pneumothorax requiring intercostal catheter	291	3.0
Assisted ventilation through endotracheal tube	67	0.7		Any body cavity surgical procedure	226	2.4
Procedures to the uterus or pelvic vessels with red cell transfusion	64	0.7		Pneumonia	192	2.0
Eclampsia	48	0.5		Resuscitation	137	1.4
Acute abdomen	41	0.4		Hypoxic ischemic encephalopathy	135	1.4
Obstetric embolism	33	0.3		Seizures	125	1.3
Acute renal failure	30	0.3		Chronic respiratory disease originating in perinatal period	103	1.1
Obstetric shock	21	0.2		Bacterial meningitis	83	0.9

© 2023 Jairam JA et al. *JAMA Network Open.*

Mothers in the second pregnancy (N = 95,617)	No. of events	Rate per 1000 births		Newborns in the second pregnancy (N = 95,605)	No. of events	Rate per 1000 livebirths
Intrapartum hemorrhage with red cell transfusion	21	0.2		Necrotising enterocolitis	79	0.8
Cerebrovascular diseases: subarachnoid & intracranial hemorrhage, cerebral infarction, stroke	12	0.1		Birth trauma	67	0.7
Placental abruption with coagulation defect	10	0.1		Grade 3 or 4 intraventricular haemorrhage	41	0.4
Pulmonary, cardiac, and CNS complications of anaesthesia during pregnancy, the puerperium, or labour and delivery	8	0.1		Periventricular leukomalacia	19	0.2

© 2023 Jairam JA et al. *JAMA Network Open.*

eTable 4. Risk of Preterm Birth Between $20^{0/7}$ to $36^{6/7}$ Weeks' Gestation Arising in the Infant's Birth Admission of the Second Pregnancy, Comparing Infants Whose Mother Moved From a Neighborhood Income Quintile 1 (Q1) to any Higher Neighbourhood Income Quintile (Q2-Q5) Between her First and Second Consecutive Births (any Upward Mobility) vs. Those With no Upward Mobility

Data are limited to nulliparous women who were initially residing in a lowest income Q1 neighbourhood at their first birth, and who had a second birth also in Ontario. Births include all singleton hospital livebirths or stillbirths at $20^{0/7}$ to $42^{0/7}$ weeks' gestation in Ontario, Canada, 2002 to 2019.

**Neighbourhood income mobility between births**	**No. with preterm birth (rate per 1000 births)**	**Unadjusted relative risk (95% CI)[a]**	**Adjusted relative risk (95% CI)[a,b]**	**Adjusted absolute risk difference, (per 1000 births, 95% CI)[a,b]**
No upward mobility (N = 53,409)	3256 (61.0)	1.00 (Reference)	1.00 (Reference)	0.0 (Reference)
Any upward mobility (N = 42,208)	2415 (57.2)	0.94 (0.90 to 0.98)	0.95 (0.92 to 0.99)	-2.9 (-5.1 to -0.6)

[a]Using an adapted approach to logistic regression analysis.[42]

[b]Adjusted for maternal age at the second birth hospitalization (15-19, 20-29, 30-39, 40-50 years); birth interval (< 18, 18-36, 37-60, 61-119, ≥ 120 months); maternal world region of origin (Canada, Caribbean, East Asia and Pacific, Latin America, Middle East and North Africa, South Asia, Sub-Saharan Africa, and, Western Nations and Europe); pre-pregnancy hypertension within 1 to 365 days before the second birth hospitalization; pre-pregnancy diabetes within 1 to 365 days before the second birth hospitalization; and gestational diabetes identified at the second birth hospitalization.

© 2023 Jairam JA et al. *JAMA Network Open.*

eTable 5. Risk of Preterm Birth Between $20^{0/7}$ to $36^{6/7}$ Weeks' Gestation Arising in the Infant's Birth Admission of the Second Pregnancy, by Degree of Upward Mobility From a Neighbourhood Income Quintile 1 (Q1) to a Higher Neighborhood Income Quintile (Q2/3 or Q4/5) Between the First and Second Consecutive Birth vs. Those With no Upward Mobility

Data are limited to nulliparous women who were initially residing in a lowest income Q1 neighbourhood at their first birth, and who had a second birth also in Ontario. Births include all singleton hospital livebirths and stillbirths at $20^{0/7}$ to $42^{0/7}$ weeks' gestation in Ontario, Canada, 2002 to 2019.

**Degree of neighbourhood income mobility between births**	**No. with preterm birth (rate per 1000 births)**	**Unadjusted relative risk (95% CI)[a]**	**Adjusted relative risk (95% CI)[a,b]**	**Adjusted absolute risk difference, (per 1000 births, 95% CI)[a,b]**
No upward mobility (N = 53,409)	3256 (61.0)	1.00 (Reference)	1.00 (Reference)	0.0 (Reference)
Moved from a Q1 to a Q2/3 neighbourhood (N = 27,735)	1641 (59.2)	0.97 (0.93 to 1.01)	0.97 (0.93 to 1.01)	-1.7 (-4.4 to 0.88)
Moved from a Q1 to a Q4/5 neighbourhood (N = 14,473)	774 (53.5)	0.88 (0.83 to 0.93)	0.90 (0.85 to 0.96)	-5.9 (-9.3 to -2.6)

[a]Using an adapted approach to logistic regression analysis.[42]

[b]Adjusted for maternal age at the second birth hospitalization (15-19, 20-29, 30-39, 40-50 years); birth interval (< 18, 18-36, 37-60, 61-119, ≥ 120 months); maternal world region of origin (Canada, Caribbean, East Asia and Pacific, Latin America, Middle East and North Africa, South Asia, Sub-Saharan Africa, and, Western Nations and Europe); pre-pregnancy hypertension within 1 to 365 days before the second birth hospitalization; pre-pregnancy diabetes within 1 to 365 days before the second birth hospitalization; and gestational diabetes identified at the second birth hospitalization.

© 2023 Jairam JA et al. *JAMA Network Open.*

eTable 6. Risk of Severe Maternal Morbidity or Maternal Mortality (SMM-M) Arising in the Mother's Birth Hospitalization of the Second Pregnancy, or up to 42 Days Thereafter, Comparing Women who Moved From a Neighbourhood Income Quintile 1 (Q1) to any Higher Neighborhood Income Quintile (Q2-Q5) Between Their First and Second Consecutive Births (any Upward Mobility) vs. Those With no Upward Mobility, Presented by Maternal Immigrant Status

Data are limited to nulliparous women who were initially residing in a lowest income Q1 neighbourhood at their first birth, and who had a second birth also in Ontario. Births include all singleton hospital livebirths or stillbirths at $20^{0/7}$ to $42^{0/7}$ weeks' gestation in Ontario, Canada, 2002 to 2019.

**Immigrant status**	**Neighbourhood income mobility between births**	**No. with SMM-M (rate per 1000 births)**	**Unadjusted relative risk (95% CI)[a]**	**Adjusted relative risk (95% CI)[a,b,c]**
Non-immigrant women	*No upward mobility (N = 33,283)*	457 (13.7)	1.00 (Reference)	1.00 (Reference)
	Any upward mobility (N = 28,299)	352 (12.4)	0.91 (0.82 to 1.00)	0.86 (0.77 to 0.95)
Immigrant women	*No upward mobility (N = 20,126)*	254 (12.6)	1.00 (Reference)	1.00 (Reference)
	Any upward mobility (N = 13,909)	155 (11.1)	0.88 (0.75 to 1.02)	0.84 (0.72 to 0.98)

[a]Using an adapted approach to logistic regression analysis.[42]

[b]Adjusted for maternal age at the second birth hospitalization (15-24, 25-34, 35-50 years); birth interval (<18, 18-36, 37-60, 61-119, ≥120 months); maternal world region of origin for immigrant women (Caribbean, East Asia and Pacific, Latin America, Middle East and North Africa, South Asia, Sub-Saharan Africa, and, Western Nations and Europe); pre-pregnancy hypertension within 1 to 365 days before the second birth hospitalization; pre-pregnancy diabetes within 1 to 365 days before the second birth hospitalization; and gestational diabetes identified at the second birth hospitalization.

[c]p-value 0.76 for the interaction of immigrant status*neighbourhood income mobility between births.

© 2023 Jairam JA et al. *JAMA Network Open.*

eTable 7. Risk of Severe Neonatal Morbidity or Neonatal Mortality (SNM-M) Arising in the Infant's Birth Admission of the Second Pregnancy, or up to 27 Days Thereafter, Comparing Infants Whose Mother Moved From a Neighborhood Income Quintile 1 (Q1) to any Higher Neighbourhood Income Quintile (Q2-Q5) Between her First and Second Consecutive Births (any Upward Mobility) vs. Those With no Upward Mobility, Presented by Maternal Immigrant Status

Data are limited to nulliparous women who were initially residing in a lowest income Q1 neighbourhood at their first birth, and who had a second birth also in Ontario. Births include all singleton hospital livebirths at $20^{0/7}$ to $42^{0/7}$ weeks' gestation in Ontario, Canada, 2002 to 2019.

**Immigrant status**	**Neighbourhood income mobility between births**	**No. with SNM-M (rate per 1000 livebirths)**	**Unadjusted relative risk (95% CI)[a]**	**Adjusted relative risk (95% CI)[a,b,c,d]**
Non-immigrant women	*No upward mobility (N = 33,283[e])*	1875 (56.3)	1.00 (Reference)	1.00 (Reference)
	Any upward mobility (N = 28,293)	1443 (51.0)	0.91 (0.86 to 0.95)	0.89 (0.85 to 0.93)
Immigrant women	*No upward mobility (N = 20,126[e])*	842 (41.8)	1.00 (Reference)	1.00 (Reference)
	Any upward mobility (N = 13,909)	582 (41.8)	1.00 (0.92 to 1.09)	0.98 (0.91 to 1.06)

[a]Using an adapted approach to logistic regression analysis.[42]

[b]Adjusted for maternal age at the second birth hospitalization (15-24, 25-35, 35-50 years); birth interval (< 18, 18-36, 37-60, 61-119, ≥ 120 months); maternal world region of origin for immigrant women(Caribbean, East Asia and Pacific, Latin America, Middle East and North Africa, South Asia, Sub-Saharan Africa, and, Western Nations and Europe); pre-pregnancy hypertension within 1 to 365 days before the second birth hospitalization; pre-pregnancy diabetes within 1 to 365 days before the second birth hospitalization; gestational diabetes identified at the second birth hospitalization; and any structural congenital anomaly diagnosed in the second infant's birth hospitalization.

[c]p-value 0.30 for the interaction between immigrant status*neighbourhood income mobility between births.

[d]The number of stillbirths were excluded in the analyses, but included in this table, to avoid back calculating small cell counts fewer than 6 persons.

© 2023 Jairam JA et al. *JAMA Network Open.*
