# Supplementary material for: Neighborhood Income Mobility and Risk of Neonatal and Maternal Morbidity
Source: JAMA Netw Open. 2023 May 23;6(5):e2315301. doi: 10.1001/jamanetworkopen.2023.15301 (PMC10208146; doi:10.1001/jamanetworkopen.2023.15301)
Supplement: Supplement 2. — Data Sharing Statement [file jamanetwopen-e2315301-s002.pdf]

Data Sharing Statement

Jairam. Neighborhood Income Mobility and Risk of Neonatal and Maternal Morbidity. *JAMA Netw Open*. Published May 23, 2023. doi:10.1001/jamanetworkopen.2023.15301

Data
Data available: No
